# Transarticular external fixation versus deltoid ligament repair in treating SER IV ankle fractures: a comparative study

**DOI:** 10.1186/s12891-019-2840-5

**Published:** 2019-10-18

**Authors:** Bohua Li, Shanxi Wang, Zhengdong Zhang, Hai Yang, Jun Li, Qin Li, Lei Liu

**Affiliations:** 1 Department of Orthopedics, The First Hospital of Lanzhou University, 1# West Donggang Road, Lanzhou, 730000 People’s Republic of China; 2 Department of Orthopedics, West China Hospital, Sichuan University, 37# Wainan Guoxue Road, Chengdu, 610041 People’s Republic of China

**Keywords:** Transarticular external fixation, ORIF, Deltoid ligament repair, Supination-external rotation, Ankle fracture

## Abstract

**Background:**

The topic that whether the injured deltoid ligament should be repaired when associated with ankle joint fractures is still discussed. The objective of this study was to compare the clinical effect of open reduction and internal fixation (ORIF) with deltoid ligament repair (DLR) or transarticular external fixation (TEF) in treating supination-external rotation type IV (SER IV) ankle fractures.

**Methods:**

Between January 2012 and December 2015, 43 patients were diagnosed as SER IV ankle fractures, 20 underwent ORIF and transarticular external fixation (TEF) without DLR (group 1), 23 were treated with ORIF and DLR (group 2). The pre- and post-operative radiographic examination were performed, the American Orthopaedic Foot and Ankle Society (AOFAS) ankle-hindfoot score, the visual analog scale (VAS), the Medical Outcomes Short Form 36-item questionnaire score (SF-36), and the ankle range of motion (ROM) were used for functional evaluation.

**Results:**

In both groups, the three scores improved significantly after surgery, but there was no significant difference between the two groups. At 6 weeks after surgery, patients in group 2 had better ankle ROM than group 1 (29.35 ± 2.033 vs. 40.35 ± 3.550, *P* <  0.001), but there was no difference at 12 months postoperatively. No cases of bone nonunion or post-traumatic arthritic changes were seen during the follow-up. Patients in group 1 required a shorter time to achieve fracture union than patients in group 2.

**Conclusions:**

ORIF with TIF is an optional strategy to manage SER IV ankle fractures as it achieves comparable functional results to ORIF with DLR. It also allows patients to start relatively earlier weight-bearing and may promote fracture union.

## Background

The deltoid ligament is an important structure to stabilize the ankle and maintain the normal anatomical position of the talus, inappropriate treatment for the ruptured deltoid ligament may cause ankle instability and traumatic arthritis [1–4]. Supination external rotation type IV (SER IV) ankle fractures are one of the most common and unstable fractures in the clinical which need surgical treatment [3, 5–9]. When the medial malleolus is intact, this type of fractures represents the ones with the injury of the deltoid ligament (AO/OTA classification 44-B3.1). Whether the ruptured deltoid ligament should be repaired or not is still controversy.

In recent years, suture-anchor was widely used for patients treated with deltoid ligament repair (DLR) [9–11], and plaster casts were most often used in treatment without DLR. Previous studies had shown that transarticular external fixators might raplace the plaster casts well by offering the opportunity for self-healing of deltoid ligament and allowing early weight bearing [12–14]. Our previous study had also shown that open reduction and internal fixation (ORIF) with transarticular external fixation (TEF) but no DLR could achieve satisfactory outcomes in treating SER IV ankle fractures [15].

Up to now, studies about ORIF, TEF but no DLR versus ORIF and DLR in the treatment of SER IV ankle fractures are rare. In this study we focused to compare the clinical and radiographic outcomes of these two methods.

## Methods

### Patients

This study was approved by the internal research board of our hospital. We retrospectively reviewed patients who were diagnosed as SER IV ankle fractures (AO/OTA classification 44-B3.1), they underwent surgical treatment between January 2012 and December 2015. The inclusion criteria were as follows: patients underwent ORIF with TEF or ORIF with DLR, pre- and postoperative X-rays evaluation, a preoperative magnetic resonance imaging (MRI) and three-dimensional computed tomography (CT), patients greater than 16 years old, and at least 12 months follow-up. The exclusion criteria included medial malleolar fracture with deltoid ligament rupture, multiple fractures, concomitant ligament injuries, open fractures, old fractures, pathological fractures, severe systemic illness, preoperative ankle dysfunction.

A total of 745 ankle fractures treated operatively were identified initially. 584 were excluded because of multiple and open fractures, 23 old fractures were also excluded, 78 were excluded because of no MRI examination, 17 were excluded because of loss of follow-up. Finally, 43 patients were incorporated into the study cohort, among which 20 were treated with ORIF and TEF (group 1), 23 were treated with ORIF and DLR (group 2). The patients’ basic information of the two groups was similar (Table 1).

**Table 1:** Comparison of the baseline data of the patients between group 1 and group 2

	Group 1(*n =* 20)	Group 2 (*n =* 23)	*P* value*
Age (years)	39.60 ± 14.420	39.22 ± 13.487	0.929
Gender (male/female)	12/8	13/10	0.818
Affected side (left/right)	7/13	9/14	0.780
Hospitalization time (days)	10.20 ± 2.067	10.09 ± 1.443	0.835
Follow-up duration (months)	29.35 ± 3.617	28.96 ± 3.994	0.738
Mechanism of injury (%)			0.604
Road traffic accident	9 (45%)	12 (52.2%)	
Simple fall	6 (30%)	8 (34.8%)	
Sports	5 (25%)	3 (13%)	

Group 1 (ORIF with TEF), and Group 2 (ORIF with DLR), *Independent *t* test or chi-square test. The *P* values shown are for inter-group comparisons

### Surgical technique

All surgeries were performed under general anesthesia by a senior surgeon. For patients in both groups, firstly the fibula fracture was exposed and the fibular rotation and length was restored, then an appropriate plate and screws were used to fixed the fibula. When the fragment was larger than 25% of the articular surface, the posterior malleolar fracture was exposed, reduced and fixed with one or two screws. The syndesmosis was assessed intraoperatively by an external rotation stress test [16, 17]. If medial clear space widened more than 5 mm continuously, syndesmotic fixation was placed.

**Fig. 1:**
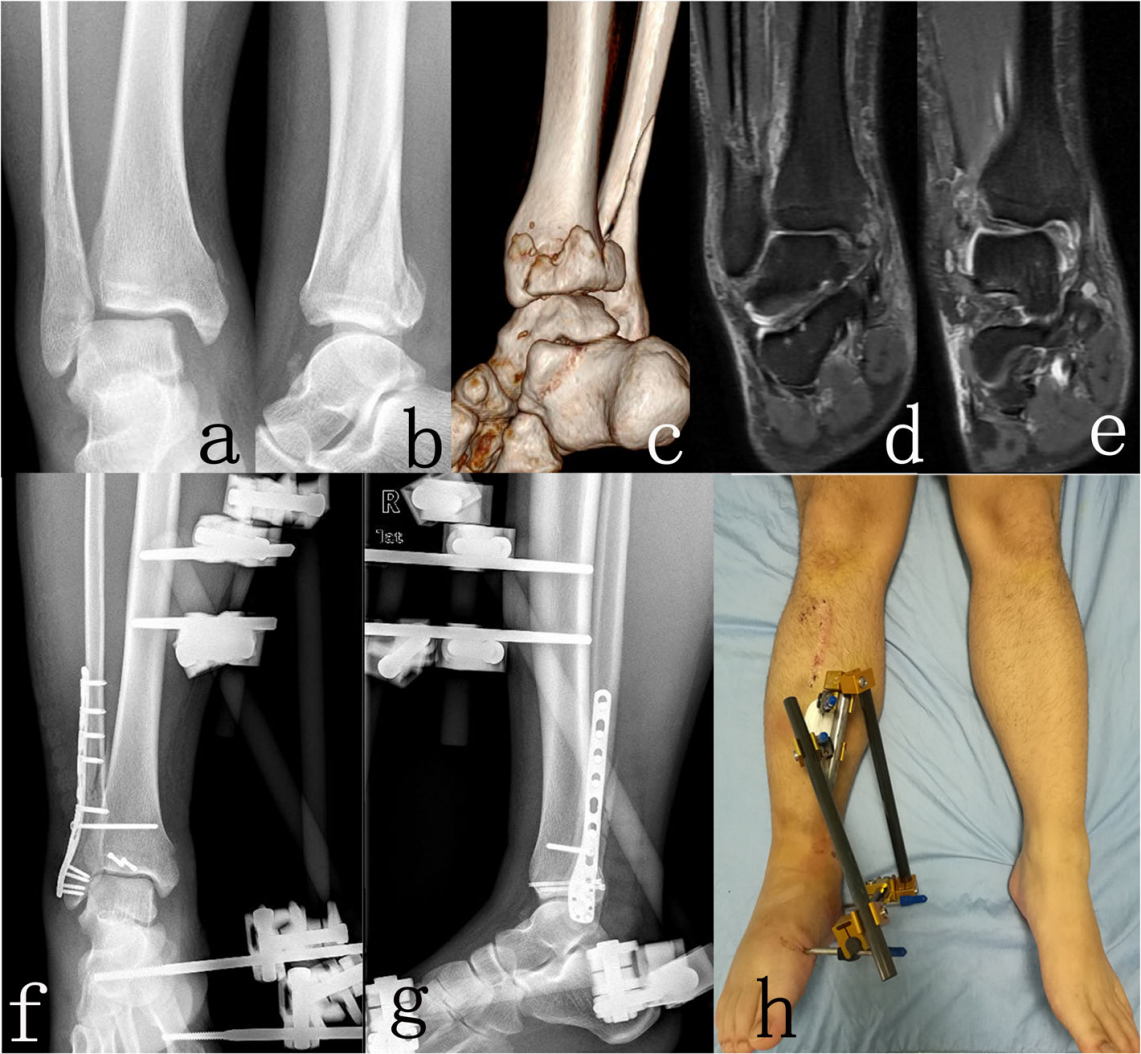
Imaging studies of a 24 years patient with SER IV ankle fractures (AO/OTA classification 44-B3.1) underwent ORIF with TEF. **a**-**c** Preoperative X-rays and three-dimensional CT. **d**, **e** Preoperative MRI showing deltoid ligament injury. **f**, **g** Postoperative anteroposterior and lateral X-rays. **h** Postoperative appearance of the affected extremity

Next, for patients in group 1, the deltoid ligament was not repaired. Schanz nails were respectively settled into the tibia, the calcaneus and the first metatarsus. The transarticular external apparatus were then installed and adjusted after ORIF, and the ankle was maintained at the neutral position (Fig. 1).

**Fig. 2:**
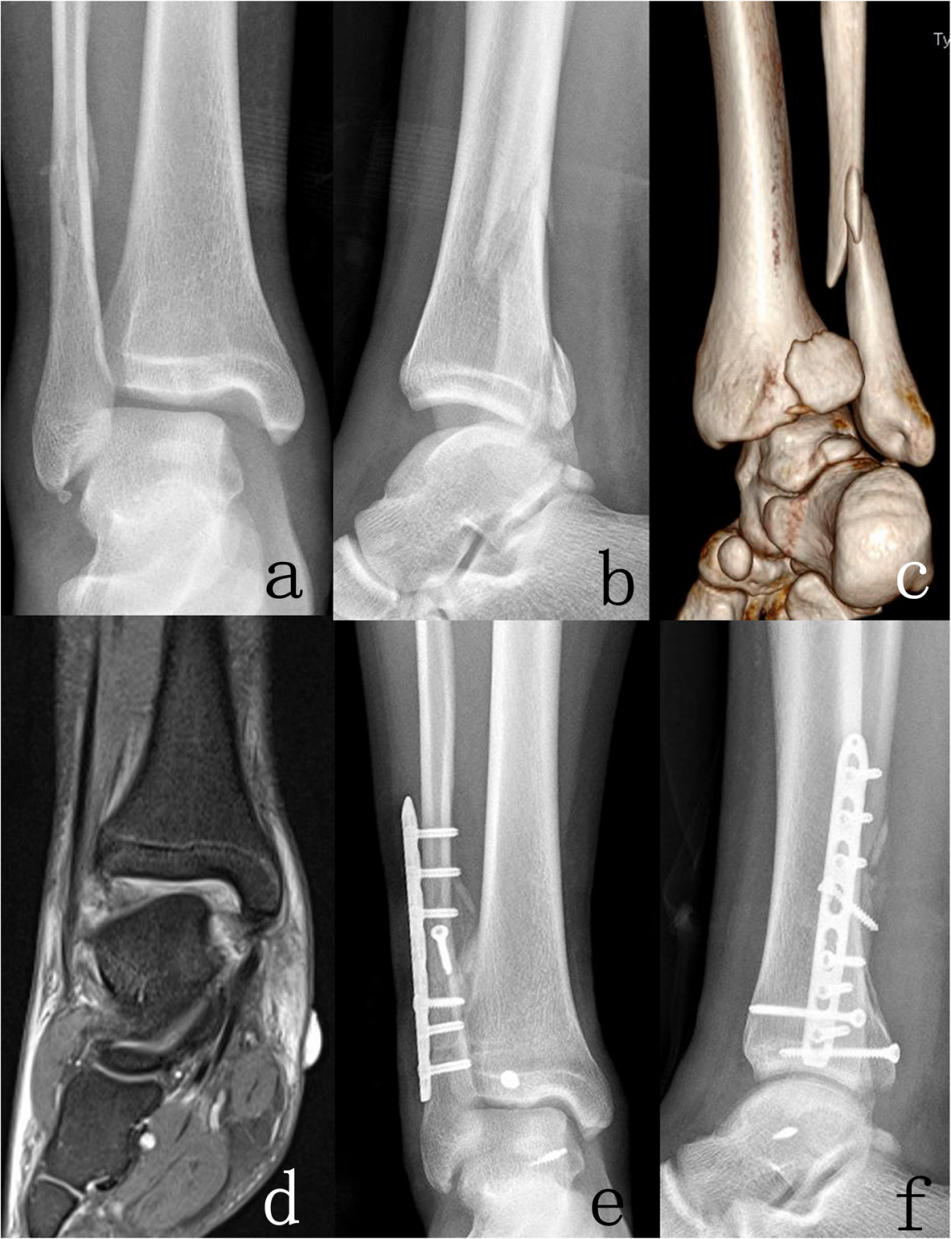
Imaging studies of a 17 years patient with SER IV ankle fractures (AO/OTA classification 44-B3.1) underwent ORIF with DLR. **a**-**c** Preoperative X-rays and three-dimensional CT. **d** Preoperative MRI showing deltoid ligament injury. **e**, **f** Postoperative anteroposterior and lateral X-rays

For patients in group 2, after adequate syndesmotic reduction and fixation, an external rotation stress test [11] was performed. The deltoid ligament was repaired if the lateral talar tilt increased or persistent medial space widened more than 5 mm. The ruptured deltoid ligament was exposed, then the superficial and deep layers were respectively repaired with sutures from the suture anchor which was inserted in the medial malleolus or medial talus (Fig. 2).

### Postoperative care

For patients in group 1, they started functional training of the lower extremity soon after operation, and began to do the ankle exercise when the external apparatus was removed at 6 weeks postoperatively. For patients in group 2, the leg was protected in a short plaster cast for 6 weeks. Ankle functional training was initiated when the pain had relieved enough. In group 1, partial weightbearing was instructed at 2 weeks postoperatively, in group 2, this time was 6 weeks. Full weightbearing was allowed when the X-ray film showed sufficient fracture healing.

### Outcome assessment

The pre- and postoperative ankle X-ray films were reviewed to observe fracture healing and diagnose post-traumatic arthritis. We also recorded other complications and patients’ inconvenience in daily life. The American Orthopedic Foot and Ankle Society (AOFAS) ankle-hindfoot score [18], the visual analog scale (VAS) [19], the Medical Outcomes Short Form 36-item questionnaire score (SF-36, [20], and the ankle range of motion (ROM) were used for functional evaluation. To avoid examiner bias, we chose two surgeons who did not participate in all patients’ surgeries to perform clinical evaluations and postoperative follow-up.

### Statistical analysis

Statistical analyses were performed with SPSS Statistics 24.0 software (SPSS Inc., Chicago, Illinois). Normality was tested using the Kolmogorov-Smirnov test. The chi-square test or Fisher exact test was used to compare categorical data. Differences between groups were evaluated by using the independent t test for normally distributed continuous data and the Mann-Whitney U tests for skewed data. The paired t test was used to compare intra-group continuous variables with normal distribution, and Wilcoxon rank test for continuous variables without normal distribution. All tests were 2-tailed. A *P* value < 0.05 was considered statistically significant.

## Results

### Baseline characteristics

The baseline characteristics of the patients were showed in Table 1. 43 patients met all exclusion and inclusion criteria. There were 20 patients in group 1 (8 males [40%] and 12 females [60%]) and 23 patients in group 2 (10 males [43.5%] and 13 females [56.5%]). With the numbers available, significant difference in patients’ age, gender, affected side, hospitalization time and follow-up duration between the two groups was not seen (*P* > 0.05). The main cause was road traffic accident (45 and 52.2% respectively), followed by falls (30 and 34.8% respectively) and sports (5 and 3%).

### Clinical outcomes

The clinical outcomes between the two groups were listed in Table 2. Patients in group 2 needed longer operative time than patients in group 1 (124.5 ± 6.403 min, 154.78 ± 8.995 min, *P* <  0.001). The perioperative blood loss of patients in group 2 was more than that of group 1 (81.96 ± 8.855 ml, 65.35 ± 6.385 ml, P <  0.001).

**Table 2:** Comparison of clinical outcomes between group 1 and group 2

	Group 1 (*n =* 20)	Group 2 (*n =* 23)	*P* value*
Operation time (min)	124.5 ± 6.403	154.78 ± 8.995	< 0.001
Blood loss (ml)	65.35 ± 6.385	81.96 ± 8.855	< 0.001
AOFAS score			
Preoperative	56.45 ± 4.359	58.04 ± 3.890	0.212
Final follow-up	91.30 ± 3.658	92.13 ± 3.494	0.451
VAS score			
Preoperative	5.50 ± 1.100	4.96 ± 1.107	0.115
Final follow-up	1.80 ± 1.056	1.70 ± 1.063	0.749
SF-36 score			
Preoperative	57.35 ± 3.990	58.09 ± 4.067	0.553
Final follow-up	78.60 ± 8.684	79.57 ± 7.216	0.693
Fracture union time (week)	8.95 ± 1.986	10.70 ± 2.548	0.017
Ankle ROM (6 weeks post-op)	29.35 ± 2.033	40.35 ± 3.550	< 0.001
Ankle ROM (12 months post-op)	59.65 ± 3.031	60.43 ± 3.273	0.422
Complications			
Infection	1 (5%)	2 (8.7%)	1.000
Nonunion	0 (0)	0 (0)	
Delayed union	0 (0)	2 (8.7%)	0.491
Arthritis	0 (0)	0 (0)	
Inconvenience in daily life			
Extremity cleaning	5 (25%)	19 (82.6%)	< 0.001
Clothing	17 (85%)	8 (34.8%)	0.002

The average AOFAS scores increased significantly in both groups, from 56.45 ± 4.359 to 91.30 ± 3.658 in group 1, and from 58.04 ± 3.890 to 92.13 ± 3.494 in group 2. The average VAS scores in both groups decreased significantly, from 5.50 ± 1.100 to 1.80 ± 1.056, and from 4.96 ± 1.107 to 1.70 ± 1.063, respectively. The average SF-36 scores in both groups increased significantly, from 57.35 ± 3.990 to 78.60 ± 8.684, and from 58.09 ± 4.067 to 79.57 ± 7.216, respectively. Nevertheless, with the numbers available, significant difference for preoperative or postoperative AOFAS, VAS and SF-36 scores between the two groups was not seen (*P* > 0.05).

During the follow-up, patients in group 1 spent less time to achieve fracture union (8.95 ± 1.986 weeks, 10.70 ± 2.548 weeks, *P* = 0.017) than patients in group 2. At 6 weeks postoperatively, the total arc of motion was 29.35 ± 2.033 degrees in group 1 compared with 40.35 ± 3.550 degrees in group 2 (*P* <  0.001). There was no difference between the 2 groups at 12 months postoperatively (59.65 ± 3.031 degrees, 60.43 ± 3.273 degrees, *P* = 0.422). In both groups, ankle instability was not seen during follow-ups, no evidences showed bone nonunion or post-traumatic arthritic changes at the last follow-up, delayed union occurred to two patients (8.7%) in group 2. Pin site infection occurred to one patient (5%) in group 1, and two patients (8.7%) in group 2 appeared superficial wound infections. They were healed after wound dressing. Their main complaints were inconvenience of clothing (17 [85%] in group 1 and 8 [34.8%] in group 2) and extremity cleaning (5 [25%] in group 1 and 19 [82.6%] in group 2).

## Discussion

The deltoid ligament is an important structure to limit the anterior and posterior translation of the talus and restrain talar abduction, with a relative contribution ranging from 50 to 80% [2, 21].

The acute deltoid ligament injury is commonly associated with ankle joint fractures. There are two most commonly used classification systems, the Lauge–Hansen, and Danis–Weber (AO/OTA) systems. The former relates the suspected mechanism of injury, the latter is based on the location of fibular fracture with respect to syndesmosis. According to the Lauge–Hansen classification, SER IV ankle fractures with an intact medial malleolus represent the ones with a deltoid ligament injury and corresponds to the type B fracture in the Danis–Weber classification (AO/OTA classification type 44-B3.1). They are one of the most common and unstable fractures in the clinical which need surgical treatment [3, 5–9]. Whether the ruptured deltoid ligament should be repaired or not is still controversy.

Many studies suggested that it was not necessary to repair the ruptured deltoid ligament in ankle fractures [1, 6, 22–25]. In Baird and Jackson’s research [22], 24 patients were diagnosed as ankle fractures with the deltoid ligament rupture, 21 patients with no DLR reached a good or excellent rate of 90%, the other 3 were treated with DLR, but 2 of them had unfavorable results. In de Souza’s study [23], 22 patients were diagnosed as the deep deltoid ligament rupture, all underwent ORIF and no DLR, all patients had satisfactory outcomes. Stromsoe et al. [24] designed a RCT, symptoms and clinical findings during follow-up showed no differences between two groups. Harper [25] reported 36 patients treated without DLR, the results showed no morbidity or evidence of ligamentous instability, he suggested that when the fibula was anatomic fixed and the medial space was maintained, DLR was not necessary.

However, some studies suggested that repair of the deltoid ligament could reduce long-term complications, patients may develop chronic ankle instability without DLR [7, 21, 25, 26]. Jones and Nunley [7] designed a retrospective comparative study including 27 patients, 12 were treated with lateral malleolus ORIF and DLR, the author concluded that repairing the deltoid ligament at the time of lateral malleolus fixation demonstrates subjective, functional, and radiologic outcomes for bimalleolar equivalent ankle fractures. Zhao et al. [21] reported 74 ankle fractures with deltoid ligament rupture, 20 patients were treated with DLR, this comparative study showed that DLR could decrease the widen medial clear space and malreduction rate. Johnson and Hill [26] reported 30 patients, treated with lateral malleolus ORIF, no DLR, the final outcome was poor in 41% of them.

As mentioned before, our previous study had shown that ORIF with TEF could achieve satisfactory outcomes in treating SER IV ankle fractures [15]. The present study retrospectively analyzed the results of 43 patients with SER IV ankle fractures who received ORIF with TEF (group 1) or ORIF with DLR (group 2).

For functional recovery and pain relieving, in both groups, AOFAS, SF-36, and VAS achieved satisfactory results after surgery, and with the numbers available, the difference between the two groups was not significant for all the three indexes. As for the total dorsiflexion/plantarflexion arc of motion, at the time of 6 weeks after surgery, patients in group 2 got significantly improved ankle ROM than patients in group 1. We considered that in the early stage, the ankle was fixed by transarticular external fixator and could not start early exercise, it brought unfavorable ankle functional results. When the fixator was removed after 6 weeks postoperatively, patients in group 1 could start ankle mobilization. Although patients in group 2 toke the exercise much earlier, there was no difference at 12 months postoperatively compared with patients in group 1. No cases of bone nonunion or post-traumatic arthritic changes were seen during the follow-up. So, patients who underwent ORIF with TEF could achieve comparable functional results and pain relieving to those who underwent ORIF with DLR.

According to biomechanical and animal studies, early weight bearing protocols is beneficial that it could optimize fracture healing [27]. For ankle fracture, early weight bearing after ORIF is feasible biomechanically without loss of reduction or fixation failure [28]. Dehghan et al. [16] designed a multicenter RCT, 110 patients with an unstable ankle fracture underwent ORIF, they were randomly divided into two groups, in the early weightbearing group, patients started full weightbearing at 2 week after operation, in the late weightbearing group, patients kept nonweightbearing for 6 weeks. The author recommended early postoperative weightbearing because it brought early improved functional outcome and did not increase complication rate. Papachristou et al. [29] designed a prospective study and suggested that early weight bearing could facilitate recovery and promote fracture union after ORIF for posterior malleolar fracture. In the present study, as ORIF and TEF could provide rigid stability and support, patients in group 1 spent less time to start weightbearing and achieve fracture union.

Both methods brought inconvenience to patients’ daily life. A majority of patients in group 2 complained that it was weary to repeat the process of taking on and off the plaster cast when cleaning the affected lower extremity. In group 1, the external fixator provided an open space that did not cover the skin, few patients thought that it was a little difficult to keep the skin clean around the external apparatus. It could also be noticed that most patients in group 1 thought it inconvenient for clothing because special trousers were needed to cope with the external apparatus, few patients in group 2 thought it difficult to wear trousers when the plaster cast was not removed.

There were several limitations in the present study. This was a retrospective study, and it was not randomized when assigning patients to different groups. We just analyzed SER IV fractures without other types of ankle fractures combined with deltoid ligament injury, the sample size was relatively small. Prospective comparative studies involving more samples are necessary for better illustration. However, in our study, the baseline data of the two groups were similar, most patients achieved satisfactory results, this study could provide reference for the management of SER IV fractures which have not been reported before. Regardless of fixation strategy, the ultimate goal is to have patients quickly and painlessly return to their preinjury activities and minimize the incidence of postoperative traumatic arthritis [5, 11, 30].

## Conclusion

In conclusion, compared with ORIF with DLR, ORIF with TEF but no DLR is an optional strategy to manage SER IV ankle fractures (AO/OTA classification 44-B3.1) as it results in favorable postoperative functional recovery and pain relieving. It also allows patients to start relatively earlier weightbearing and may promote fracture union.

## Data Availability

The data of this study were real and were performed using SPSS Statistics version 24.0 software (SPSS Inc., Chicago, Illinois). All of the data are available in contact with the correspondence author.

## Abbreviations

AOFAS: American Orthopaedic Foot and Ankle Society
DLR: Deltoid ligament repair
ORIF: Open reduction and internal fixation
ROM: Range of motion
SER: Supination-external rotation
SF-36: Medical Outcomes Short Form 36-item questionnaire
TEF: Transarticular external fixation
VAS: Visual analogue scale
